# Diffusion-Weighted Magnetic Resonance Imaging Findings of Kidneys with Obstructive Uropathy: Differentiation between Benign and Malignant Etiology

**DOI:** 10.1155/2014/980280

**Published:** 2014-02-09

**Authors:** Tugce Ozlem Kalayci, Melda Apaydin, Fitnet Sönmezgöz, Sinan Çalık, Melike Bedel Koruyucu

**Affiliations:** ^1^Department of Radiology, Izmir Ataturk Training and Research Hospital, 35160 Izmir, Turkey; ^2^Department of Radiology, Firat University Medical Faculty, 23100 Elazig, Turkey; ^3^Department of Statistic, Faculty of Science, Fırat University, 23100 Elazig, Turkey

## Abstract

*Purpose*. In this study, we aimed to evaluate the capability of diffusion-weighted magnetic resonance imaging (DWI) in differentiation between benign and malignant etiology of obstructive uropathy. *Materials and Methods*. DWI was performed in 41 patients with hydronephrotic kidneys and 26 healthy volunteers. MR imaging was performed using a 1.5 T whole-body superconducting MR scanner. The signal intensities of the renal parenchyma on DWI and apparent diffusion coefficient (ADC) maps were noted. DWI was performed with the following diffusion gradient *b* values: 100, 600, and 1000 s/mm^2^. A large circular region of interest was placed in the corticomedullary junction of the kidneys. For statistical analysis, the independent-samples *t* test was used. *Results*. The mean renal ADC values for *b*100, *b*600, and *b*1000 in hydronephrosis patients with benign and malignant etiology and the healthy volunteers of the control group were analysed. ADC measurements of renal parenchyma in all hydronephrotic kidneys with benign and malignant etiology were found to be statistically low compared to those of normal kidneys (P < 0.05). *Conclusions*. There were significant differences in the ADC values of obstructed kidneys compared to those of normal kidneys. Obstructed kidneys with malignant etiology had lower ADC values for *b*1000 compared to obstructed kidneys with benign etiology, but these alterations were statistically insignificant.

## 1. Introduction

Diffusion-weighted magnetic resonance imaging (DWI) is used to show the Brownian motion of the spins in biologic tissues and can be used to differentiate between normal and abnormal tissue structures. The apparent diffusion coefficient (ADC), as the main quantitative parameter used to interpret DWI, combines the effects of capillary perfusion and water diffusion in the extracellular extravascular space [1]. DWI has been extensively used in neuroradiology. The applications of DWI in abdominal disease have lagged behind those of neurologic applications because DWI of abdominal organs is much more difficult to perform because of physiologic motion artefacts and the heterogeneous composition of the organs [2]. With the advent of echoplanar imaging (EPI) in conjunction with breath-holding, DWI of the abdomen has become possible with fast imaging times, minimizing the effect of gross physiologic motion from respiration and cardiac movement. The kidney is an interesting organ to measure ADC values because of its high blood flow and water transport functions. With its complex anatomic structure and physiology, the kidney is extremely challenging for DWI [3, 4]. Obstructive uropathy can occur due to some benign and malignant conditions. The benign and malignant causes of hydronephrosis might result in different diffusion characteristics in the affected renal parenchyma. To date, no papers have been published on DWI in obstructive uropathy patients for discrimination between benign and malignant etiology. The purpose of this study was to evaluate the capability of DWI in differentiation between benign and malignant causes of obstructive uropathy.

## 2. Materials and Methods

The Institutional Ethics Committee reviewed and approved the study protocol, and informed consent was obtained from all volunteers and patients.

### 2.1. Patients

Forty-one patients with chronic hydronephrotic kidneys detected by ultrasound (US) imaging participated in the study. There was a history of obstructive uropathy longer than 6 weeks in all patients. Twenty-six patients (20 male and 6 female; mean age 58.3 ± 17.8; age range 24–90 years old) with benign etiology and 15 patients (10 male and 5 female; mean age 62.3 ± 18.1; age range 21–80 years old) with malignant etiology were included in this study. The control group consisted of 26 healthy volunteers (8 male and 18 female; mean age 49.0 ± 18.8; age range 27–65 years old) on whom was performed abdominal MRI for hepatic haemangioma. They also had no history of renal disease and had normal creatinine levels (0.7 ± 0.12 mg/dL). Seven of the 26 patients with benign etiology and seven of the 15 patients with malignancy had bilateral hydronephrosis. The 33 kidneys (30.8%) with obstructive uropathy with benign etiology exhibited benign prostatic hyperplasia (9.3%, *n* = 10); ureter stone (4.6%, *n* = 5); renal calculus within the renal pelvicalyceal system (7.4%, *n* = 8); and narrowness of the ureter secondary to retroperitoneal fibrosis (9.3%, *n* = 10). The twenty-two kidneys (20.6%) with obstructive uropathy with malignant etiology exhibited bladder cancers (8.4%, *n* = 9); colon cancers (1.8%, *n* = 2); cervical cancers (2.8%, *n* = 3); uterine cancers (0.9%, *n* = 1); prostate cancers (0.9%, *n* = 1); retroperitoneal tumours (1.8%, *n* = 2); and pelvic tumours (3.7%, *n* = 4). All patients with obstructive uropathy with benign and malign etiology were previously diagnosed by radiologic imaging or histopathologic study.

### 2.2. Magnetic Resonance Imaging (MRI)

MRI was performed using a 1.5 T whole-body superconducting MR scanner (General Electric Signa high-speed scanner, Milwaukee, WI, USA) equipment with high-speed gradients. A body coil was used for all images. Axial T2-weighted fat saturation spin-echo images (TE: 90, TR: 5700, slice thickness: 8 mm, intersection gap: 1.5, number of excitations: 4, and matrix size: 512 × 512) were obtained in all patients for demonstration of the pelvicalyceal system. DWI (TE: 72, TR: 8000, FOV: 30 × 30, slice thickness: 5 mm, intersection gap: 0, number of excitations: 1, and matrix size: 128 × 128) was obtained using single-shot spin-echo and echoplanar imaging (EPI) sequences with the following diffusion gradient *b* values: 100, 600, and 1000 s/mm^2^. All images were obtained without restriction of fluid intake and without breath-holding.

### 2.3. Image Analysis

**Figure 1:**
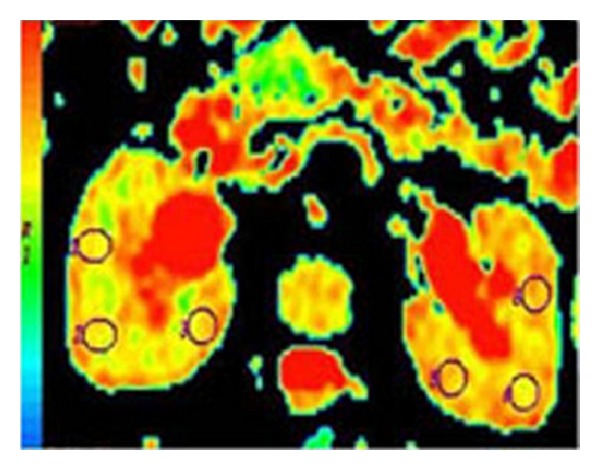
Axial ADC map calculated from echoplanar DWI of hydronephrotic kidneys with a high *b* value. The yellow-green coloration was observed significantly in the right hydronephrotic kidney to be compatible with lower ADC values. The ROIs are placed in three locations: an anterior portion, an intermediate site, and a posterior portion.

The DWI data were transferred to a workstation (Advantage Windows, software version 2.0, GE Medical Systems). Radiological analysis was performed by the same radiologist. A large circular region of interest (ROI) was placed at the corticomedullary junction for the measurement of ADC values (Figure 1). For each kidney, three ROIs were placed in the middle portion of the kidneys, which are less influenced by the perfusion effect. The mean ADC values for *b*100, *b*600, and *b*1000, with standard deviations, were calculated. ADC maps were calculated automatically with the MR system.

### 2.4. Statistical Analysis

Statistical analysis was performed with the SPSS 12.0 software package. The ADC values of the volunteers and patients with obstructed uropathy are reported as the mean ± standard deviation. The independent-samples *t* test was used to compare the parenchymal ADC values of the normal kidneys and the obstructed kidneys that had benign and malignant etiology. A *P* value of less than 0.05 was considered to indicate a statistically significant difference.

## 3. Results

**Figure 2:**
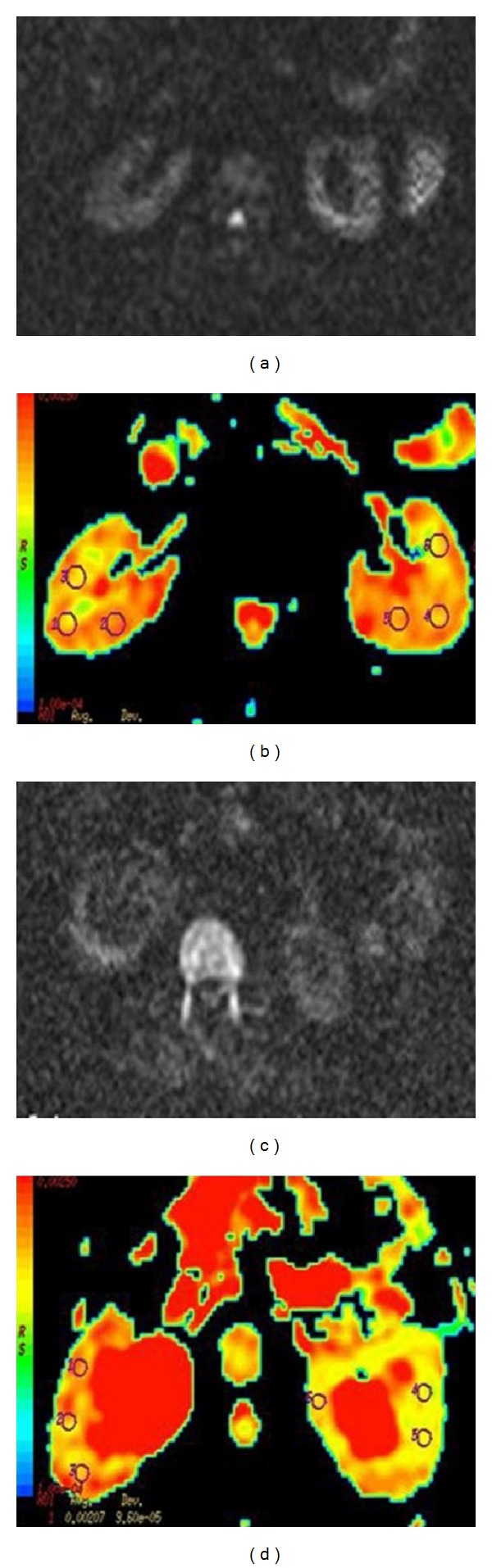
Normal kidneys ((a), (b)) and hydronephrotic kidneys ((c), (d)). Axial apparent diffusion coefficient (ADC) map calculated from echoplanar diffusion-weighted images of healthy and hydronephrotic kidneys with high *b* values at the central portion of normal, healthy kidneys ((a), (b)) and hydronephrotic kidneys ((c), (d)). A large circular region of interest (ROIs) was placed at the corticomedullary junction for the measurement of ADC values for normal and obstructed kidneys.

Significant declines were observed in renal signals with an increasing value of *b* in the obstructed kidneys. The colour change was observed on the ADC maps that were created from DW echoplanar images, depending on increasing *b* value and decreasing ADC coefficients; the colour shift from red to yellow/green was observed much more in hydronephrotic kidneys than in normal kidneys to be compatible with lower ADC values (Figure 2).

**Figure 3:**
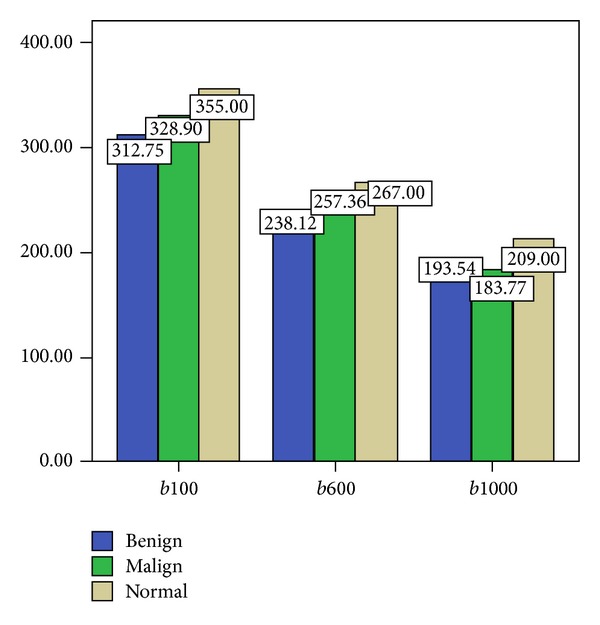
Comparison of parenchymal ADC values of obstructed kidneys with benign-malignant etiology and normal kidneys, for *b*100, *b*600, and *b*1000 (mm^2^/sn).

The mean renal ADC values for *b*100, *b*600, and *b*1000 values in patients with obstructive uropathy with benign and malignant etiology and in the healthy volunteers of the control group are summarised in Table 1. The ADC measurements of renal parenchyma in all hydronephrotic kidneys with benign and malignant etiology were found to be extremely low compared to those of normal kidneys (*P* < 0.05) (Figure 3).

There was a statistically significant difference between the ADC values of hydronephrotic kidneys with benign causes and those of normal kidneys. The mean ADC values of hydronephrotic kidneys with benign etiology were statistically significantly lower than the mean ADC values of normal kidneys for *b*100, *b*600, and *b*1000 (*P* < 0.05). The mean ADC values of hydronephrotic kidneys with malignant causes were found to be statistically significantly lower than the mean ADC values of normal kidneys for *b*100 and *b*1000 (Table 1).

In the obstructed kidneys with benign etiology, the minimum and maximum values of ADC ranged from 1.45 to 4.10 × 10^−3^. In the obstructed kidneys with malignant etiology, the minimum and maximum values of ADC ranged from 1.49 to 4.02 × 10^−3^. Obstructed kidneys with malignant etiology had lower ADC values for *b*1000 than had the obstructed kidneys with benign etiology, but these differences were statistically insignificant.

## 4. Discussion

Hydronephrosis is a common disease in urological clinical practice, which is one of the major causes of renal insufficiency and renal failure. Dilatation of the renal pelvis and calyceal system can occur even in the absence of urinary obstruction; therefore, hydronephrosis and obstructive uropathy are not interchangeable or synonymous terms. Obstructive uropathy can occur due to some benign and malignant causes. Common causes include bladder stones, kidney stones, benign prostatic hyperplasia, bladder or ureteral cancer, colon cancer, cervical cancer, uterine cancer, scar tissue that occurs inside the ureter, and problems with the nerves of the bladder. Up to now, there have been various approaches to define what obstruction really means, including US, intravenous urography, diuretic renal scintigraphy, abdominopelvic computed tomography imaging, and MRI. MRI can correctly identify the point of obstruction and the noncalculus causes of obstruction. MR excretory urography is a promising technique that affords equivalent functional and additional anatomical information to that of isotope renography [5].

DWI allows the noninvasive measurement of ADC values and, in a clinical setting, provides simultaneous information on the diffusion, and perfusion of kidneys. When applying high *b* values, the influence of perfusion is largely cancelled out, the ADC value approximates diffusion and low *b* values are influenced by both perfusion and diffusion. The lack of consensus regarding the selection of *b* values makes it difficult to compare results from different investigators and to generate standardised ADC values in disease and health [6–9]. It is also important to choose ROIs in the proper portion of the kidney. Some authors [10, 11] have reported higher values in the medulla than in the renal cortex. In our study, we did not try to evaluate ADC values in the cortex and in the medulla separately because it may be difficult and inaccurate to position the ROI cursor on the renal cortex and the medulla of the kidney separately, as pointed out by Fukuda et al. [12]. The evaluation of ADC values in the middle portion of the kidneys is thought to be less influenced by the perfusion effect. In our study, the ROI cursors were placed at the approximate level of the corticomedullary junction. In the mesorenal area, we preferred the evaluation recommended by Fukuda et al. [12].

Several studies have investigated the use of DWI for hydronephrotic kidneys. Bozgeyik et al. [13] demonstrated that an early-phase obstructed nonfunctioning kidney has statistically insignificant lower ADC values compared to the contralateral normal functioning kidney. Similarly, in the evaluation of patients with hydronephrosis, Toyoshima et al. [14] showed that hydronephrotic kidneys with moderate and severe decreases in renal function as assessed with renal scintigraphy had significantly lower mean ADC values than had hydronephrotic kidneys with maintained renal function. Thoeny et al. [3] reported DWI of the kidneys in healthy volunteers and patients with various renal abnormalities. In their study, the patients with acute ureteral obstruction DWI did not reveal any significant differences between obstructed and contralateral nonobstructed kidneys. They also demonstrated that all ADC values of the kidneys in the patients with pyelonephritis were substantially lower compared with those of the opposite side. In addition, they showed that the patients with renal failure had significantly lower ADC of the cortex and medulla than did volunteers. Verswijvel et al. [11] reported lower ADC values in affected parenchymal areas in three patients with acute pyelonephritis, in one case of pyogenic abscess, and in one patient with xanthogranulomatous pyelonephritis compared with the normal renal parenchyma. Chan et al. [15] reported concerning a set of 12 patients that the pelvicalyceal system of the hydronephrotic kidneys (66.6%, *n* = 8) was hypointense on DW images, while the pelvicalyceal system of the pyonephrotic kidneys (33.4%, *n* = 4) was markedly hyperintense, compatible with restricted diffusion. These studies highlight the potential role of renal ADC values in the evaluation of hydronephrotic kidneys.

We concluded that many pathological renal conditions, such as chronic renal failure, pyelonephritis, and obstructive disorders, decrease the ADC values of kidneys. However, to the best of our knowledge, the effect of obstructive uropathy with benign versus malignant etiology on the ADC values of kidneys has not been reported. In the present study, there was a statistically significant difference between the ADC values of patients with obstructive uropathy and those of normal, healthy volunteers, with lower ADC values for hydronephrotic kidney, compatible with previous studies in the literature. However, we did not find a statistically significant difference between the ADC values of patients of obstructive uropathy with benign versus malignant etiology. Therefore, the degree and duration of obstruction may cause diffusion restriction in renal parenchyma.

## 5. Conclusions

DWI seems to be a reliable method to differentiate normal healthy kidney from hydronephrotic kidney. On the basis of this preliminary study, this technique could be applied in the clinical area as a rapid addition to existing kidney MRI protocols and thus provide DW images of diagnostic quality as well as quantitative data regarding diffusivity. The present study reports on our initial experience with DWI of the kidneys in patients with obstructive uropathy with benign and malignant etiology in a small sample and further studies using ROIs in different locations (e.g., the renal pelvis) and larger groups of obstructive uropathy patients are warranted to assess the efficacy of DWI for the discrimination of etiology.

## Figures and Tables

**Table 1:** Comparison of ADC values of obstructed kidneys with normal kidneys.

*b* value	Obstructed kidneys with benign etiology Mean ± S.D.	Obstructed kidneys with malignant etiology Mean ± S.D.	Normal kidneys Mean ± S.D.
100*	3.12 ± 0.61 × 10^−3^**	3.28 ± 0.44 × 10^−3^**	3.55 ± 0.29 × 10^−3^
600*	2.38 ± 0.45 × 10^−3^**	2.57 ± 0.68 × 10^−3^	2.67 ± 0.49 × 10^−3^
1000*	1.93 ± 0.33 × 10^−3^**	1.83 ± 0.17 × 10^−3^**	2.09 ± 0.19 × 10^−3^

ADC average values calculated from the following *b* values: 100, 600, and 1000 s/mm^2^.

*Independent *t* test, 0.05 significance level.

**The differences are statistically significant between the groups.
